# Adopting Artificial Intelligence in Public Healthcare: The Effect of Social Power and Learning Algorithms

**DOI:** 10.3390/ijerph182312682

**Published:** 2021-12-01

**Authors:** Tara Qian Sun

**Affiliations:** Department of Digitalization, Copenhagen Business School, 2000 Frederiksberg, Denmark; qs.digi@cbs.dk

**Keywords:** artificial intelligence, learning algorithm, social power, healthcare, AI adoption, IT adoption, influencing factor

## Abstract

Although the use of artificial intelligence (AI) in healthcare is still in its early stages, it is important to understand the factors influencing its adoption. Using a qualitative multi-case study of three hospitals in China, we explored the research of factors affecting AI adoption from a social power perspective with consideration of the learning algorithm abilities of AI systems. Data were collected through semi-structured interviews, participative observations, and document analysis, and analyzed using NVivo 11. We classified six social powers into knowledge-based and non-knowledge-based power structures, revealing a social power pattern related to the learning algorithm ability of AI.

## 1. Introduction

Given the recent developments and accumulation of user data, the capability of computing has greatly improved in recent years. The continuing optimization of algorithms has resulted in a rapid increase in the development and use of artificial intelligence (AI) systems across various industries. In the healthcare sector, although the adoption rate of AI has been slow compared with other industries, there has been increasing interest from stakeholders [1,2,3]. For example, the Chinese government has proposed several policies to promote the development of AI in practice, including the Three-Year Guidance for Internet Plus Artificial Intelligence Plan [4] and the Next Generation Artificial Intelligence Development Plan [5].

We define the adoption of AI in healthcare as the stage at which a decision is made by hospitals or individual users (such as patients, doctors, and medical personnel) to adopt an AI technology. AI can be described as systems that simulate cognitive functions related to human learning, speech, and problem-solving [6]. Hence, AI technology refers to any application or device that perceives the environment in a similar manner to humans and acts to optimally achieve a goal. AI technologies include machine learning systems, deep learning systems, rule-based systems, imaging recognition systems, natural language processing, and voice recognition [7,8].

Previous information systems research has explored the adoption of technology in various sectors, including finance [9,10], universities [11,12,13], healthcare [14,15,16,17,18], and firms [19,20,21,22,23], and has investigated the factors affecting the adoption of information technologies (IT) from different perspectives, including technology diffusion and the technology acceptance model.

With respect to emerging technologies such as AI, with its human-like ability to perceive the environment and self-learn, it is crucial for information system scholars investigating AI adoption to consider its uniqueness. Learning algorithms refer to “an emergent family of technologies that build on machine learning, computation, and statistical techniques, as well as rely on large data sets to generate responses, classifications, or dynamic predictions that resemble those of a knowledge worker” [24]. Learning algorithms are crucial characteristics of AI technology that affect many aspects of work and organization, including reshaping occupational work and boundaries, providing new forms of control, and transforming expertise [24]. Learning algorithms represent unique characteristics of AI that may be linked to its adoption in the healthcare context.

However, the characteristics of healthcare settings, such as the life-saving nature of doctors’ work and their esoteric medical knowledge, have led to physicians being accustomed to working independently and autonomously [25]. Thus, it can be challenging for stakeholders such as hospital managers and IT firms to persuade medical practitioners to use new technologies. Moreover, given that doctors often have little knowledge of AI systems, attracting their attention can be difficult. Therefore, the influence of other stakeholders on the adoption of IT by doctors is an important topic.

The unique characteristics of AI and healthcare settings increase the difficulty of pinpointing the factors affecting IT adoption. Research has been conducted on the factors influencing the adoption of electronic medical records (EMR) by understanding physician and caregiver identities and perceived government influence on caregivers [25]. However, studies on the adoption of AI in the healthcare sector are lacking. To fill this gap, the present study investigated the following research question: *How does social power among various stakeholders affect IT adoption in healthcare?*

We aimed to answer this question by identifying social power strategies used by various stakeholders—patients, doctors, hospital staff, and IT firm managers and staff—and linking these social power strategies with the required level of learning ability of the AI systems adopted in three hospitals in China.

The remainder of this paper is structured as follows. In Section 2, we provide the background of the study by defining AI learning algorithms, clarifying the required learning ability of AI, and discussing the existing research on AI adoption in healthcare. Section 3 introduces the theoretical lens used by this study—social power—to analyze the empirical data and the factors influencing AI adoption in healthcare. In Section 4, we present the research setting (three hospitals that had adopted AI technologies), the data collection methods and results, and the analysis of the data. In Section 5, we present the findings of our three case studies, showing the dominant social powers related to the adoption of AI systems with high and low levels of learning algorithm ability, respectively. Section 6 summarizes the findings of the study by presenting the social power strategies that align with both high and low levels of required learning ability of AI systems. This section also highlights the study’s contributions to social power and IT adoption research. In Section 7, we summarize the main findings, present the limitations of this study, and discuss the contributions of the study to future research.

## 2. Background and Previous Research

### 2.1. Learning Algorithm Ability of AI

Learning algorithms are defined as “an emergent family of technologies that build on machine learning, computation, and statistical techniques, as well as rely on large data sets to generate responses, classifications, or dynamic predictions that resemble those of a knowledge worker” [24].

AI systems typically have various training objectives that determine the training data and algorithms used. Algorithms are a core feature of AI [2,24,26,27,28] and can influence the adoption of AI technology. However, given the low level of AI adoption in practice, particularly in the medical field in which AI use is still in the early stages, there is a lack of research on the effect of learning algorithms on AI application.

The training data used in medical AI systems vary according to the system [29,30]. For example, the training data used in receptionist robots typically include basic textual information on hospital departments, doctors, disease types, and hospital floor distributions, as well as interactions between nurses and patients at medical guidance desks that help patients interpret voice data from consultations. Voice-based EMR training data mainly include clinician–patient interaction questions and patient examination data. Diagnostic AI requires algorithm training data such as clinical medical guidelines, data from the medical literature, doctors’ voice data, medical imaging data, and case-taking data.

Data from clinical examinations, such as laboratory, imaging, and pathology results, objectively reflect patients’ conditions. Clinical case-related data include the information discussed between clinicians and patients, such as subjective descriptions of symptoms and the progress of disease, along with relevant clinical information, such as case information, laboratory results, imaging examinations, and the clinician’s own clinical knowledge. Depending on the needs of individual patients, information generated from the differential diagnosis of disease and the development of feasible treatment plans should be objectively documented in medical records of inpatients during hospitalization, as should the results of all clinical manifestations and examinations for special diseases. Once a case has been analyzed, the corresponding treatment measures are recorded. Complete case data should not only include comprehensive information about a patient’s condition but should also reflect the progress of the disease and the clinician’s treatment plan.

The quality of the case data generated by physicians, including both objective information, such as patient examination, and subjective judgments, such as condition analysis, is affected by the level of diagnosis and treatment, how comprehensively the condition has been understood, and the accuracy and comprehensiveness of data entry. Results from laboratory testing and imaging procedures, such as blood tests, liver function tests, coagulation profiles, magnetic resonance imaging (MRI), and computerized tomography (CT), are objectively generated by instruments that are not influenced by a clinician’s subjective judgment, thus can be completely preserved without human input. Therefore, from the perspective of large data, the quality of medical examination data is generally higher than that of clinical case-taking data.

Before exploring the effect of learning algorithms on AI adoption, it is necessary to define learning algorithm ability, which is the arithmetic capability required by an AI system according to its training objectives and training data. This study focused on AI learning algorithms based on training data volume, data velocity, data quality, and data variations required by AI systems.

### 2.2. Research on AI Adoption in Healthcare

Given that the use of AI in healthcare is still in its early stages, the empirical research in this field is lacking [31,32]. In addition, because the medical field involves human life and health problems, it presents unique characteristics. Until the technology is fully mature, its promotion and application will be relatively slow.

Few scholars have conducted empirical studies on the adoption of AI in healthcare. One study used Q-methodology to examine the shared beliefs and concerns of doctors and other stakeholders regarding the introduction of healthcare service robots to hospitals [33]. Another used case studies of AI use in hospitals to research how knowledge embodiment of AI affects the relationship between people and technology from the perspective of social informatics [34]. The increasing application of technology in a wide range of organizations and industries has led scholars to explore the various opportunities associated with AI, including its use in organizational decision-making and problem-solving [29], its augmenting capabilities, and its ability to replace workers [24,29]. This study aims to fill the gap in the research by exploring the factors influencing AI adoption in healthcare.

## 3. Theoretical Lens

Social power is defined as the potential influence of power rather than the actual use of power or influencing techniques by power figures over subordinates [35]. Raven and colleagues [35,36] introduced six power sources: coercive power, reward power, legitimate power, expert power, referent power, and informational power.

Coercive power refers to the threats of penalty or punishment by way of imposing unpleasant fines for noncompliance exerted by a person or a group [35,37,38]. Reward power refers to promised monetary or nonmonetary compensation offering a positive return for compliance exerted by a person or a group [35,37,38]. Legitimate power refers to the person’s right to influence others, given by a legitimate position in the organizational hierarchy or norm [35,37,38]. Expert power refers to the perception that an agent has some special or superior knowledge that possesses the expertness influence to the target [35,37,39]. Referent power refers to the person’s (target) identification with the influencing agent [35,39]. Informational power refers to the potential influence of a different perspective or knowledge that an influencing agent might impart to a target [35].

Constructs of social power have been used by information systems (IS) scholars to understand the influencing factors of IT adoption. For example, some research has studied the influence of power of managers stipulating subordinates or interns comply with behavior, and the influence of the power with which one can oblige colleagues or other organization members to assist her/him based on social responsibility norms for IT adoption in the Chinese healthcare context [38]. Moreover, social power has been studied in the context of EMR system adoption, in which harsh power and soft power were distinguished [37]–reward power, coercive power, and positional legitimacy power were treated as harsh power, while informational power and referent power were treated as soft power [37]. The results of this study show that soft power has a significant influence on indirect EMR use, and harsh power has a significant influence on direct EMR use [37].

A study of adoption of EMR in the U.S. shows that mimetic forces and normative forces have a significant influence on EMR adoption in hospitals when the government has not introduced incentive policies [40]. Following the introduction of policies by the government, coercive forces also play a significant role [40]. Second, within the hospital organization, the chief information officer (CIO) strategic leadership, top management team (TMT) attitude toward IT, and hospital climate have an obvious influence on hospital IT innovation [17]. Moreover, the capacity of a hospital to absorb and assimilate information on health information technology (HIT), the capacity of an adaptor to impart and disseminate knowledge on HIT, and the interaction between these two capacities can affect adoption [41].

## 4. Methods

### 4.1. Research Setting

To answer the research question, we used a multi-case study method to investigate three hospitals—Hospital X, Hospital Y, and Hospital Z—applying medical AI systems. Hospital X, based in Anhui, used four medical AI systems—a voice-based AI receptionist robot, a voice-based AI EMR system, an AI-assisted medical imaging system, and an AI-assisted diagnostic system. Hospital Y used two medical AI systems—a voice-based AI receptionist robot and a voice-based AI EMR system. Hospital Z also used two medical AI systems—a voice-based AI EMR system, an AI-assisted medical imaging system, and an AI-assisted diagnostic system.

The four medical AI systems adopted by the hospitals in this study were all provided by iFlytek and represent all of the medical AI systems currently offered by iFlytek. Medical AI systems provided by other IT enterprises were not included in this study. In the following discussion, we use the terms “receptionist robot”, “voice-based EMR”, “medical imaging AI”, and “diagnostic AI” to refer to the four medical AI systems. The medical AI systems adopted in each of the three cases are presented in Table 1.

IFlytek is a Chinese intelligent machines firm focusing on research on intelligent speech and language technologies, development of software and chip products, provision of speech information services, and integration of E-government systems. It was established in 1999, and is headquartered in Hefei, Anhui province [42,43]. The voice assistant technology, “the Siri of China,” is the core technology of iFlytek, and represents the top level in the world. iFlytek accounts for 70% of China’s market in voice-based AI technologies.

#### 4.1.1. Adopted Medical AI Systems

Receptionist robots are patient-centric technologies used by patients, while voice-based EMR systems are staff-centric technologies used by doctors from various hospital departments. Receptionist robots are located in the reception area of the hospital with the aim of answering patients’ questions. It is important to differentiate patient-centric technologies from staff-centric technologies because adoption of the latter is affected by different power structures.

Voice-based AI EMR system has been used at Hospital Y to help doctors and medical staff to improve the efficiency of the input of patient data, as the voice signal can be directly converted into the corresponding text through the system. In many departments of general hospitals, such as the breast surgery, orthopedic, general surgery and some other departments, the number of patients is extremely large, and doctors must write a large number of medical records each day.

Medical imaging AI systems can assist radiologists to rapidly understand patients’ clinical imaging data from procedures, such as CT and MRI scans. The use of medical imaging AI can save significant amounts of time, reduce doctors’ workloads, and help to quickly eliminate negative results [44]. Additionally, medical imaging AI uses quantitative analysis, reducing the rate of misdiagnosis. Radiologists typically use qualitative methods based on personal experience and medical knowledge to draw conclusions, which can lead to misdiagnosis or missed diagnosis for various reasons, including fatigue caused by long working hours, limited personal experience in diagnosis and treatment, and limited medical knowledge. Medical imaging AI can accurately analyze images using higher learning algorithm abilities, improving the rate of accurate disease identification and helping doctors reduce the rate of missed diagnosis and misdiagnosis [44,45].

Diagnostic AI systems can help clinicians, including family doctors, improve their diagnostic abilities and reduce the potential for misdiagnosis. Diagnostic AI systems are based on a high volume of clinical cases and medical data, including clinical voice data and clinical imaging data, and use high levels of learning ability to generate possible diagnoses according to the patient information entered by the doctor. This is especially useful for traditional and family doctors, who have relatively limited abilities in differentiating and diagnosing complex diseases and can be prone to misdiagnosis. Given their large databases, diagnostic AI systems can provide all possible differential diagnoses as well as relevant diagnostic information based on a patient’s overall condition. For example, when a traditional family doctor visits an elderly patient with hypertension and inputs the patient’s data, a diagnostic AI system can provide the risk of coronary heart disease, congenital cardiomyopathy, and pulmonary heart disease as 86%, 45%, and 30%, respectively. Diagnostic AI can provide differential diagnostic information for various conditions and play a highly effective auxiliary role in differential diagnosis, which is especially useful for general practitioners and significantly reduces the rate of misdiagnosis. Additionally, diagnostic AI systems enable the equitable provision of high-quality medical resources based on large data, enabling patients in different locations, especially remote areas where medical resources are scarce, to enjoy the benefits [46].

#### 4.1.2. Case of Hospital X, Anhui

Hospital X, located in Hefei in Anhui province, is a level 3-A provincial university hospital. Table 2 provides a ranking list of Chinese public hospitals, and a comparation of the three hospitals: Hospital X, Y, and Z. Hefei is the capital of Anhui province, located in the eastern part of China and, in 2018, had a total population of more than 8 million people.

**Figure 1:**
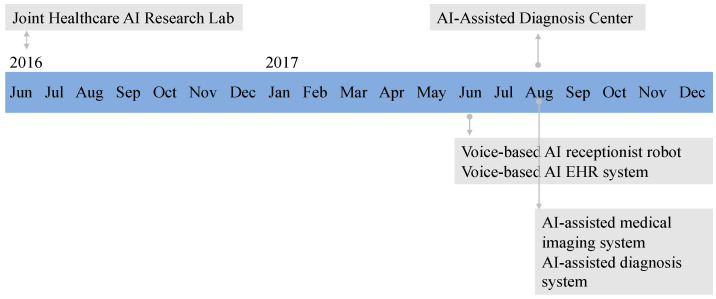
Timeline of the introduction of four artificial intelligence (AI) systems.

Hospital X used four AI systems provided by iFlytek: a receptionist robot, a voice-based EMR system, a medical imaging AI system, and a diagnostic AI system. Hospital X is a university hospital with a strong interest in research. In collaboration with iFlytek, Hospital X founded a joint healthcare AI research center in 2016 and an AI-assisted diagnostic center in 2017. In June 2017, the voice-based AI receptionist robot and voice-based AI EMR system were introduced at Hospital X of Anhui. In August 2017, the AI-assisted medical imaging system and the AI-assisted diagnosis system were introduced at Hospital X. Figure 1 illustrates the timeline of the two joint healthcare AI research centers and the four AI systems used by Hospital X.

#### 4.1.3. Case of Hospital Y, Beijing

Hospital Y is a military hospital located in Beijing, China. Founded in 1953, it is aimed at building a first-class modern research-based hospital and following the principle of ‘being the leading force in the army, a first-class hospital in China and a high-level medical care provider in the world. Since October 2017, Hospital Y has used two AI systems provided by iFlytek: a receptionist robot and a voice-based EMR system.

The reason for choosing Hospital Y is that it is a military hospital and located in Beijing, which is the center of politics in China. Compared with Hospital X of Anhui, Hospital Y is organized differently, and the adoption of AI was influenced by different power resources. As a military hospital, Hospital Y is highly centralized, which can provide a different and diverse research context for studying the adoption of AI in Chinese healthcare. Moreover, it provides a suitable context to investigate the adoption of an emerging new technology from the perspective of social power. In other words, investigating how different kinds of powers such as legitimate power or referent power from leaders or supervisors influence users’ adoption behavior in this military context hospital is important.

#### 4.1.4. Case of Hospital Z, Anhui

Since April 2018, community Hospital Z has used two AI systems provided by iFlytek—a voice-based EMR system and a diagnostic AI system.

Hospital Z is a local community hospital located in Heifei, which is similar to a clinic but has more departments; however, it is not like a general or a special hospital that deals with complex cases. The main role of Hospital Z is to provide basic medical information and treatment for local patients, and refer complex patients to a general or a special hospital. The doctors who work at Hospital Z are not as competent as doctors from large comprehensive hospitals because of the lack of complex clinical cases. Among the three cases that adopted AI systems provided by iFlytek, this is the most recent. The reasons for this include (1) Hospital Z is a community hospital, and does not have the ability to conduct research and development on AI systems; (2) as a community hospital, it only has basic clinical data with limited local patients, and therefore, both the data quality and data size are not sufficient to train AI systems; and (3) Hospital Z needed to wait for a developed AI system to be introduced in its hospital.

**Figure 2:**
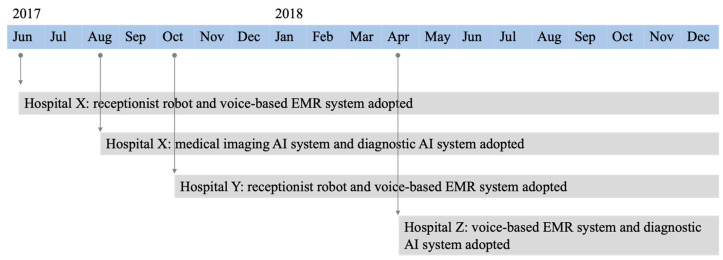
Timeline of medical artificial intelligence (AI) systems adopted in the three cases.

The timeline of the adoption of the medical AI systems for all three cases is shown in Figure 2.

### 4.2. Data Collection

We adopted a multiple case study approach [47] to answer the research question. Primary data were gathered from semi-structured interviews and direct observation of the use of AI by doctors and patients in hospitals. Secondary data were drawn from conference presentations and public interviews of iFlytek’s CEO and other experts.

Semi-structured interviews were conducted with six groups of stakeholders: (1) doctors from each case hospital, (2) hospital managers from each case hospital, (3) top managers from iFlytek, (4) staff from iFlytek working on-site at each case hospital, and (5) patients at the hospitals, (6) hospital service staff employed to help patients.

Data collection from the three cases occurred simultaneously. In total, 29 interviews were conducted with 24 informants. The overall interview time was 900 min, with each interview lasting from 10 to 90 min. The interviews conducted with the managers from iFlytek provided information that was applicable to all three hospitals. All interviews were recorded with the interviewees’ permission, transcribed immediately following each interview, and were translated from Mandarin Chinese to English. Table 3 provides an overview of the interview data for the three cases, showing interview duration, interview numbers, and informant numbers for each stakeholder groups. The asterisk (*) denotes the shared interview minutes, interview numbers, and informant numbers for the three cases.

Participant observation was used to reveal contextualized and otherwise inaccessible data to understand the tacit knowledge shared between organizations [48]. The observations were of daily working scenarios, case presentations, and demonstrations at each of the three hospitals and at iFlytek. In total, 10 observations with a total duration of 470 min were conducted. Table 4 provides an overview of participant observation data.

In addition, we analyzed one interview, four conference presentations, one public online interview, three official news stories, one television dialogue, and one academic presentation concerning the adoption of AI in hospitals as perceived by iFlytek and Hospital X. Analysis of secondary data sources was aimed at triangulating the data generated from interviews with IT firm manager on their views of the factors influencing adoption of the AI systems used in the case hospitals with previously published data. 

### 4.3. Data Analysis

Each dataset was analyzed to answer the research question. Using the concept of social power as a sensitizing device [35,36], we aimed at identifying and classifying the views of stakeholders (hospital managers, doctors, IT staff, and hospital staff) on the factors influencing AI technology adoption.

All interviews were recorded, transcribed, and translated from Mandarin Chinese to English. With the support of NVivo 11 software, interview transcriptions were coded using three rounds. The first round of coding was aimed at inductively identifying specific actions, activities, or behaviors that decreased user resistance and facilitated use of AI [49]. In the second round of coding, we deductively regrouped the first-order codes into more abstract second-order codes that synthesized the specific actions, activities, or phenomena into six distinct concept groups—coercive power, legitimate power, reward power, expert power, referent power, and informational power [35,36]. In the third round of coding, the six concept groups were inductively abstracted and distinguished into two social power strategies—a knowledge-based power strategy and a non-knowledge-based power strategy. The interview data, observation data, and secondary data were analyzed using the same procedure. Interview data coding examples are shown in Table 5.

The interview transcriptions were coded in three rounds. The first round of coding aimed at identifying specific behaviors or actions that different stakeholders interpreted to facilitate AI adoption in an inductive fashion (Strauss & Corbin, 1998). The second round of coding re-grouped the first-order codes into more abstract second-order codes that synthesized the interpreted behaviors and actions into six distinct concept groups—coercive power, legitimate power, reward power, expert power, referent power, and informational power (Raven, 2008; Raven et al., 1998). In the third round of coding, the six concept groups were inductively abstracted and distinguished into two strategies—directive strategies and participative strategies. 

For example, the first-order coding, the results of specific behaviors or actions interpreted by different stakeholders included punished by leaders, reporting the usage data of AI during the department weekly meeting, rewarding doctors who were actively participating in the use and development of AI, use of AI by colleagues, updating the system and providing expert solutions to problems faced by doctors, encouraging knowledge sharing of AI to doctors, and providing training to doctors. 

In the second-order coding, Raven’s (2008) six social powers was used to deductively re-group the first-order coding; for example, “punished by leaders” was re-grouped to coercive power and “reporting the usage data of AI during the department weekly meeting” was re-grouped to legitimate power. Finally, in the third-order coding, the six powers were grouped into two strategy groups; for example, coercive power and reward power were assigned to mandatory strategy.

Analysis of the data drawn from direct observations of AI systems being used in practice or being developed, designed, and improved helped provide additional insights into the factors influencing the use of AI in healthcare as perceived by patients, doctors, and IT staff. Similarly, analysis of the ten secondary data sources provided additional insights into the influencing factors and background information of adopted AI systems by the IT firm iFlytek. The analysis of participative observation notes and pictures and the secondary data helped to provide additional insights and a background against which to assess the relevance of findings from the interviews. Secondary data are available upon request.

## 5. Findings

Our findings from the three case studies reveal the power structures among stakeholders that influenced the adoption of AI in healthcare. Two power structures were identified: a knowledge-based power structure and a non-knowledge-based power structure. Knowledge-based power structures are social powers related to knowledge and skills, such as expertise in AI, and include expert power, informational power, and referent power. Non-knowledge-based power structures refer to social powers relating to organizational behaviors, including reward power, coercive power, and legitimate power, and have no relation with personal knowledge and skills. Moreover, our findings show the relationship between the power structure and the learning algorithm ability of the AI system (see Table 6).

As discussed in this paper, learning algorithm ability is defined as the arithmetic capability needed by an AI system according to its training objectives and training input data. For example, medical imaging and diagnostic AI systems have higher learning algorithm abilities than receptionist robot and voice-based EMR systems for several reasons. First, with respect to data volume, medical imaging and diagnostic AI systems use imaging data with larger data volumes and require higher algorithm capabilities. Second, with respect to data velocity, the acquisition of medical imaging data is slower. Third, with respect to data quality, medical imaging systems require large volumes of training data related to clinical case-taking, which are lower in quality compared with data related to clinical examinations (as discussed at Section 2.2). Finally, from the perspective of data diversity, medical imaging and diagnostic AI systems require more types of data and more complex data; thus, they need higher learning algorithm abilities. Table 7 shows the distribution of the four AI systems by training data and learning algorithm ability.

### 5.1. AI with Low Learning Algorithm Ability

Findings from the three cases show that the power structure of stakeholders involved with AI systems with low learning algorithm abilities is knowledge-based, primarily informational power and expert power. We investigated two AI systems—receptionist robot and voice-based EMR. Voice-based EMR systems are staff-centric, while receptionist robots are patient-centric. The power structures should be discussed separately as the stakeholders involved with these two AI systems are different. We report the findings for each of the AI technologies below.

#### 5.1.1. Patient-Centric AI

Among the investigated AI systems, the receptionist robot was the only patient-centric AI in which the users were patients rather than doctors or other hospital staff. First, our findings show that a knowledge-based power structure was effective in facilitating patient use and that the knowledge and skills of different stakeholders influenced usage. For example, as seen in the case of Hospital X, both IT staff and hospital service staff affected patients’ usage by regularly providing updates to the receptionist robot (i.e., expert power), while hospital staff provided tacit knowledge and usage skills (i.e., informational power). One patient commented, “I saw this robot; however, I don’t know how to use it [at first]. This guy [the gatekeeper at Hospital X] taught me” (1P01). Our observation of how the gatekeeper helped patients use the receptionist robot (1OB05) verified this tacit knowledge. During our observation of the updating process of the receptionist robot by iFlytek employees, both professional AI algorithm knowledge and hospital information resulted in the continual improvement of the receptionist robot (1OB06). The continual updating of the receptionist robot was mentioned by patients at both Hospital X and Hospital Y as a primary reason for using the receptionist robot: “Now it [the receptionist robot] is smarter [than a few months prior], I would like to use it” (1P01) and “It makes it easier and quicker for us to see a doctor” (2P01). These comments imply that patients’ adoption behaviors are influenced by the expert knowledge of IT firm staff.

Second, patient groups tended to seek a collective identity and were influenced by other users’ opinions (i.e., referent power), as mentioned by one of our informants: “There is a long line at the reception desk [of the hospital] … I saw she is using, then I come and want to have a try”. (2P02)

In addition, besides the three knowledge-based social powers, coercive power was mentioned at Hospital X:

In order to improve the patient acceptance of the voice-based AI receptionist robot, we organized a no-nurse day activity. Every Saturday, all nurses who are usually working at the hospital lobby are no need to work. Instead, AI receptionist robots will work at the hospital lobby to service patients. Of course, we [on-site staff] will have one or two people watching … We suggested this activity and got supported by hospital managers.

(4IE02)

This shows that patients were influenced by activities organized by both the IT firm and the hospital. However, this only occurred at Hospital X. Hospital Y is a military hospital in which there was a low level of cooperative activities to publicly promote the receptionist robot, and it was difficult for IT providers to conduct product promotion activities.

#### 5.1.2. Staff-Centric AI

Voice-based EMR systems were used by doctors in all three cases. The findings of our study showed that a knowledge-based power structure (informational power and expert power) was effective for AI systems with low learning algorithm abilities. Informal chats and frequent communications between IT staff and doctors (informational power) positively influenced the adoption of voice-based EMR. This is demonstrated by the following comment from an iFlytek informant who was responsible for adoption of the voice-based EMR at Hospital Z:

First of all, when you have free time, come to chat with them more often, ask them whether they have been used recently, find out why they are useless, or ask them what problems they found when they were used, so that we can solve them in time and improve the quality of products.

(3IT01)

Although the IT firm provided doctors with opportunities to be trained in a variety of ways, non-adoption was mentioned by doctors from Hospital X: “At first we felt awkward to use it. After our training, we feel unable to adapt to this method of work, so we still tend to adopt the original methods of work” (1HD03). After some false starts, doctors enthusiastically began to use the AI system. An important factor was the strategy used by IT staff to help doctors solve problems in a timely fashion. One doctor interviewee commented:

Last year [for several months at the start of the year], they [iFlytek] had an employee stay at my office working together with me … She [the on-site person from iFlytek] had a notebook and made notes every time I had problems using it. She showed me how to use [it] and helped me a lot on daily use … For example, if I work from 7 am to 12 pm, she will stay with me from 7 am to 12 pm … Then I started the real use of it.

(1HD04)

Especially at the process of using [voice-based], we will stay at the hospital and watching their [doctors] work, in which we talk to doctors about the problems they faced during the use of our product. We took notes carefully and will come back to our employees to find the solutions.

(1IT02)

The IT firm provided training (i.e., informational power) at Hospital X and Hospital Y to help doctors use the AI systems. One doctor commented:

They [iFlytek] have trainings for us, especially at the beginning … it is very helpful … and anytime they have updates of the system, they will have a small-scale training for us to let us know the new functions and changes.

(1HD04)

In addition, AI technology-related knowledge shared via social media between IT staff and doctors improved doctors’ understanding and adoption of AI. As mentioned by a hospital manager from Hospital Z:

“I have their WeChat [social media] and can see their posts about the news of their products and AI frequently … Then I know that this is a good and advantaged product which I would like to try”.

(3HM01)

Compared with Hospital X and Hospital Z, Hospital Y had infrequent use of informal methods such as casual visits from IT staff to inform doctors about updates to AI system or sharing of AI knowledge and news with doctors via social media (e.g., WeChat). While these methods were considered effective by staff in Hospitals X and Z, Hospital Y’s status as a military hospital may have affected the use of informal methods, as implied by one respondent:

“Most of us use formal means, such as from the initial bidding to the introduction of AI equipment later, mostly through formal meetings”.

(2HM01)

Informational power was evident in Hospital X and Hospital Z, but not in Hospital Y, which only involved expert power such as training, talks, and expert seminars. As one doctor interviewee mentioned, “IT firm people seldom come directly to our office … Usually we meet at meetings” (2HD01).

### 5.2. AI with High Learning Algorithm Ability

The data from our three cases show that the power structures among stakeholders using AI with high learning algorithm abilities were non-knowledge-based, primarily reward power and legitimate power. We investigated two staff-centric AI systems—diagnostic AI and medical imaging AI.

Rewarding doctors who frequently used AI and provided valuable feedback to improve the AI system was mentioned by one interviewee from the IT firm:

At the first year, we organized marketing activities to facilitate the doctors’ use to AI … We set first prize, second prize and third prize to users who can contribute to the usage or improvement … and counted by usage count and valuable feedback.

(1IT03)

Doctors did not comment on whether they were influenced by rewards but did mention that neither hospital managers nor IT firm staff used coercive power such as penalties for failing to use the diagnostic or medical imaging systems: “Even if we don’t use it, nobody will punish us” (1HD01). Our observation of doctors using diagnostic and medical imaging AI systems showed that, although they can provide doctors with fast diagnostic advice, the use of these two systems was still at an early stage. Because both systems required doctors to input data manually, data input was a time-consuming process (1OB02, 1OB03). The diagnostic system and the original EMR system of the hospital were independent of each other and had not yet achieved data sharing. Doctors were not only required to input data into the EMR system but also into the diagnostic AI system, resulting in a doubling of the workload and a reluctance by doctors to use the diagnostic AI system [1OB03]. As one of the respondents from Hospital Z pointed out, “If I have many patients [appointments], I would not use diagnosis AI system” [3HD01].

Despite the fact that doctors were unwilling to use the system because of the extra workload, hospital managers exerted a legitimate power influence over doctors. Specifically, this involved holding weekly meetings for doctors to report on their use of diagnostic and medical imaging AI systems, including the frequency of use and any problems encountered. This kind of influence on doctors arising from leadership demands for reporting had a positive effect on doctors’ use of AI:

Last year [for several months at the start of the year], every week we had a regular meeting with doctors and hospital managers. During the meeting, the head of each department from the hospital attended. And, doctors need to report the use condition of AI products during the weekly meeting … If they didn’t use, then they have nothing to report … This gives doctors pressure to use the AI product.

[1IT04]

In addition, findings showed that when leaders modeled the use of medical imaging AI systems and assigned tasks developed jointly with the IT firm to subordinates, this effectively promoted the adoption of AI technology by young doctors:

My supervisor has research collaboration with iFlytek, and they will study how to better train the medical imaging AI system in order to improve its accuracy. What I and other [young] doctors usually do is collect raw materials according to my supervisor’s requirement and provide to machine [AI] to learn … Data annotation is also done by us.

[1HD01]

Hospital Y did not use any AI technology with high learning algorithm abilities provided by the IT firm. This may be because AI technologies with high learning algorithm abilities must be developed jointly with hospitals, and the professional knowledge of doctors is required to help improve accuracy. Hospital Z did not use a medical imaging AI system, which was mainly attributable to its hospital status. As a community hospital, the ability to diagnose complex conditions was limited; hence, there was no medical imaging department in the hospital.

## 6. Discussion

### 6.1. Summary of Findings

This empirical study of three hospitals in China and four AI systems found that social power as a factor affecting AI adoption was related to the learning algorithm abilities of the AI systems. The adoption of AI systems with high learning algorithm abilities in the healthcare sector was more susceptible to non-knowledge-based power factors such as reward power and legitimate power. Such powers are typically related to personal positions or organizational behaviors and are not related to specific knowledge possessed by employees. The adoption of AI systems with low learning algorithm abilities in the healthcare sector was more easily influenced by knowledge-based power factors such as expert power, information power, and referent power. Such powers influenced the adoption of technology mainly through the professional knowledge and skills of influencers, including tacit and explicit knowledge.

**Figure 3:**
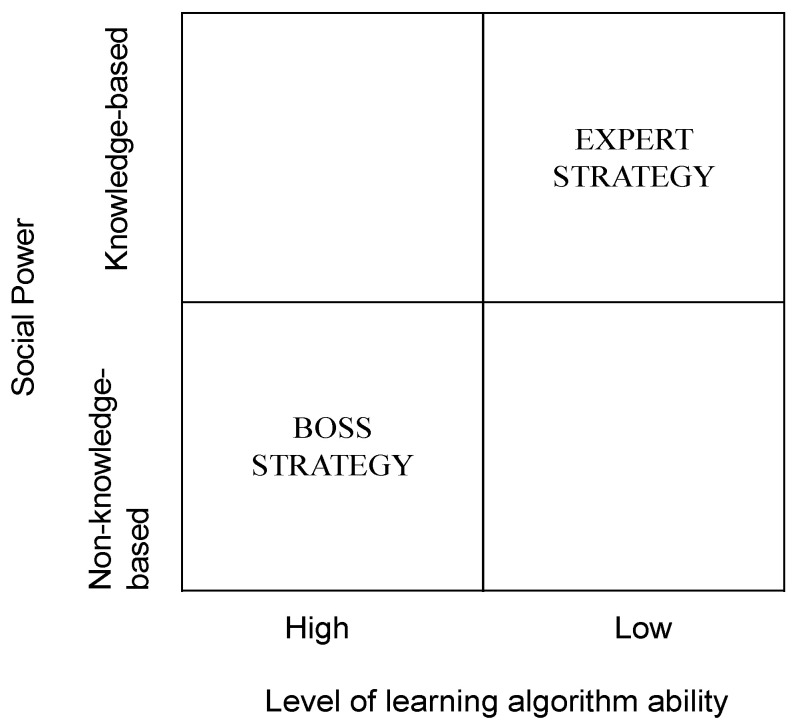
Power strategy matrix of AI adoption in healthcare.

These findings allow us to theorize about the social powers influencing the adoption of AI in the healthcare sector by considering the unique features of both AI and healthcare settings. We used the terms “expert strategy” and “boss strategy” to describe the broad power strategies involved with AI adoption and to further understand the relationship between the two overarching social power structures and the learning algorithm ability of the AI technology. Figure 3 illustrates the overall power strategies involved with AI adoption in healthcare. It positions the four investigated AI technologies, providing a better understanding of the adopted AI systems in healthcare for future studies.

First, our study showed that both knowledge-based power and non-knowledge-based power among stakeholders can influence the adoption of AI in the healthcare sector. Previous researchers have studied hard and soft power influences on IT adoption in healthcare [37,38]. The findings of our study show the consistent role of knowledge-based social power in influencing AI adoption. Social powers based on knowledge and skills pertaining to AI had a different effect to non-knowledge-based powers. Therefore, in our study, we classified the six social powers proposed by Raven and Bertram [35] into knowledge-based and non-knowledge-based power structures and further studied the influence of these power structures on the adoption of AI in the healthcare sector.

Second, our study showed that the learning algorithm ability of the AI system should be considered when studying the adoption of AI. The adoption of AI systems with high and low learning algorithm abilities is influenced by different social powers. For example, the adoption of medical imaging AI systems is mainly influenced by reward power and legitimate power, while the adoption of voice-based EMR systems is mainly influenced by information power and expert power, such as informal training and knowledge sharing by IT firms via social media. In addition, our study found that the adoption of patient-centric AI systems (e.g., receptionist robots) is affected by coercive power, even though this system has a low learning algorithm ability. When studying the influence of social power on the adoption of AI, users should be differentiated according to their knowledge base. For example, patients are vastly different from doctors in terms of their medical knowledge. As found in previous research, doctors represent a stakeholder group with unique personal identity characteristics [25].

Third, our study showed the relationship between social power and the learning algorithm ability of AI. We used the terms “expert strategy” and “boss strategy” to describe the overall power strategies influencing AI adoption. Expert strategy refers to the phenomenon in which knowledgeable persons have a significant effect on AI adoption. In this situation, adopted AI systems are “lower” in intelligence, making them easier to accept and understand by users (i.e., doctors). Once the IT firm has provided relevant information on the use of an AI system (i.e., knowledge-based power), adopters can easily understand and learn the system.

**Figure 4:**
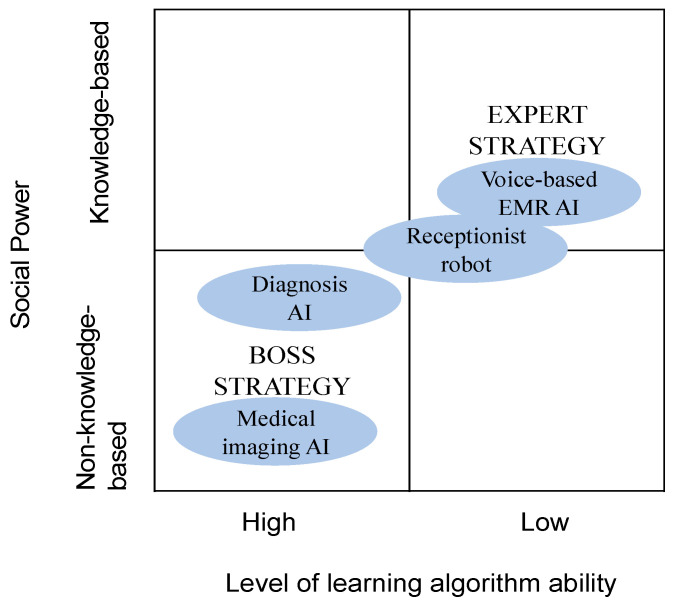
Understanding AI adoption and power strategy.

In contrast, boss strategy refers to the phenomenon in which individuals in high positions have a significant effect on AI adoption. In this situation, adopted AI systems are “higher” in intelligence, requiring doctors as medical experts to be involved in the design and development of the AI system. As shown in Figure 4. Therefore, the relationship between adopters and IT providers becomes more collaborative. As mentioned by one IT firm manager:

I think the relationship between some of the doctors and us is both friends and enemies. When we interviewed the doctors in the CT department, we also said that we are both friends and enemies. At first, we worked together to do some work, then we helped you [doctors] improve [work efficiency], and then I [doctors] helped you [IT firm] mark some data, so that you [IT firm] can constantly improve the accuracy [of medical imaging AI system].

(IT02)

Because collaboration with IT firms typically involved hospital managers rather than doctors (who were somewhat independent), the factors influencing doctors’ adoption of AI mainly originated from their supervisors.

### 6.2. Contributions to Theory

The findings of our study contribute to the existing social power theory as well as the research on the factors influencing IT adoption in organizations. The findings also answer our research question: How does social power among various stakeholders affect IT adoption in healthcare?

Our study extends the existing understanding of social power theory in two ways. First, our study identified knowledge as an important issue, with social powers being differentiated into two groups—knowledge-based social power and non-knowledge-based social power. This is important for the adoption of AI based on learning algorithms. Our findings have generated a number of factors for future research on the adoption of emerging technologies with the ability to learn. The knowledge construct of social power is important to understand, and different types of social power can influence the adoption of IT in different ways. For emerging technologies with learning abilities, knowledge plays a more important role than ever before.

Second, our study linked the categories of social powers involved in AI adoption to the level of learning algorithm ability. Through this empirical finding, we have contributed to social power theory and aim to inspire further studies using social power theory to analyze the adoption of technologies, especially for emerging technologies with the ability to learn. It is important for researchers to consider the learning abilities of emerging technologies when studying their adoption. Further, when using social power theory to study the influence of power on the adoption of technology, the characteristics of different users and organizations should be considered. As our findings show, patients are different from doctors, as are the social powers involved.

Using the example of AI in healthcare, our study contributes to the research on the adoption of emerging technologies, which may assist other information system scholars to better understand the implementation of emerging technologies, which is different to that of traditional technologies. Although AI is only one example of emerging technologies, it is representative because of its learning ability. The learning algorithm ability of a technology should be considered when studying not only its adoption but also its post-implementation by future scholars.

Finally, our study contributes to the previous research on the adoption of technology in the healthcare context by exploring the use of new AI technologies in healthcare. Previous research on technology adoption in healthcare has mainly focused on technologies such as EMR and health IT. However, with the development of emerging technologies such as AI, their adoption in the healthcare sector should be studied by scholars. Although the adoption of emerging technologies in healthcare has been left behind compared with other sectors, stakeholders such as doctors, hospital managers, government policymakers, and patients understand the importance for hospitals to adopt emerging technologies to improve the accuracy and efficiency of medical services.

### 6.3. Managerial Implications

Our study has implications and recommendations for IT firm managers with knowledge of AI systems and their learning algorithm abilities. These findings suggest that IT firm managers should consider different types of learning algorithms when collaborating with hospitals and use different strategies of power. For example, for AI systems with low levels of learning ability, IT firms should consider influencing doctors by sharing AI knowledge through informal communications, such as social media (e.g., WeChat), or solving problems by visiting doctors at their place of work. Moreover, our findings show that the boss strategy is suitable for the adoption of diagnostic and medical imaging AI systems, while the expert strategy is suitable for the adoption of voice-based EMR and receptionist robot systems. For example, regarding diagnostic and medical imaging AI, the IT firm should consider increasing their collaboration with hospitals because hospital managers play a more important role than IT firms in promoting the adoption of AI.

## 7. Conclusions

In summary, our study found two social power strategies—the expert strategy and the boss strategy—that are suited to AI systems with different levels of learning algorithm ability when adopting AI in healthcare.

Although our study has made several contributions to both research and practice, it has some limitations. First, in our three cases studies, only Hospital X used all four AI technologies, while the other hospitals only adopted two of the AI systems. This limits our findings from the data. In future, we call for more studies on the adoption of AI in hospitals using multiple AI systems.

Second, we only investigated one patient-centric AI system (the receptionist robot), while the other three AI systems were staff-centric technologies. Therefore, this makes our findings less robust for understanding the differences between patient-centric and staff-centric AI systems. In future, studies should be carried out that focus on and compare patient-centric and staff-centric AI systems.

Third, given that we used social power as our theoretical lens, we did not consider governmental institutional factors. In China, healthcare is a public service that is influenced by the government sector; however, because we adopted a social power perspective, this institutional perspective was outside of the scope of this research. Therefore, we recommend that future researchers investigate government policymakers as stakeholders and explore the influence of power factors from the government sector.

## Figures and Tables

**Table 1:** Adopted artificial intelligence (AI) systems in the three cases.

Case	AI System			
	Receptionist Robot	Voice-Based EMR	Medical Imaging AI	Diagnostic AI
Hospital X	x	x	x	x
Hospital Y	x	x		
Hospital Z		x		x

**Table 2:** Description of targeting hospitals using AI systems provided by iFlytek.

Hospital No.	Hospital Attribute	Hospital Level	Hospital Rank (1st to 9th)	Hospital Reputation	Location
Hospital X	Provincial hospital	Level 3-A (San Ji Jia Deng, third-grade class-A hospital ^†^)	First	High	Hefei city, Anhui province
Hospital Y	National hospital	Level 3-A (San Ji Jia Deng, third-grade class-A hospital)	First	Top	Beijing city, Beijing
Hospital Z	Community hospital	Level 1-A (Yi Ji Jia Deng, first-grade class-A hospital)	Seventh	Low	Hefei city, Anhui province

^†^: The National Health Commission (NHC) of P.R.C evaluated Chinese hospitals as three levels—Level three, Level two and Level one from top to down. And in each level, NHC divided them to three grades which are A, B and C from top to down. Totally, there are nine levels of all hospitals. For example, the hospital of the highest standard belongs to Level 3-A.

**Table 3:** Overview of case interview data.

Stakeholders	Position	Informant Code	Interview mins	Interview N	Informant N
Informants of Hospital X					
Hospital managers	Department head	1HM01	20	1	1
Doctors	Medical doctor	1HD01	30	1	1
	Medical doctor	1HD02	20	1	1
	Medical doctor	1HD03	20	1	1
	Medical doctor	1HD04	25	1	1
Hospital service stuffs	Hospital gateman	1S01	15	1	1
Patients	Patient	1P01	10	1	1
iFlytek managers *	Vice CEO	IT01	60	1	1
	Department manager	IT02	80	1	1
	Department manager	IT03	50	1	1
On-site stuffs of iFlytek	Employee	1IT01	60	2	1
	Employee	1IT02	30	1	1
	Employee	1IT03	30	1	1
	Employee	1IT04	20	1	1
Summary of Hospital X			470	15	14
Informants of Hospital Y					
Hospital managers	Department head	2HM01	90	3	1
Doctors	Medical doctor	2HD01	60	2	1
Hospital service stuffs	Hospital gateman	2S01	15	1	1
Patients	Patient	2P01	10	1	1
	Patient	2P02	20	1	1
iFlytek managers *	Vice CEO	IT01	60	1	1
	Department manager	IT02	80	1	1
	Department manager	IT03	50	1	1
On-site stuffs of iFlytek	Employee	2IT01	40	1	1
Summary of Hospital Y			425	12	9
Informants of Hospital Z					
Hospital managers	Department head	3HM01	30	1	1
Doctors	Medical doctor	3HD01	35	1	1
iFlytek managers *	Vice CEO	IT01	60	1	1
	Department manager	IT02	80	1	1
	Department manager	IT03	50	1	1
On-site stuffs of iFlytek	Employee	3IT01	110	2	1
	Employee	3IT02	20	1	1
Summary of Hospital Z			385	8	7
Summary of three cases					
Hospital X			470 (190) *	15 (3) *	14 (3) *
Hospital Y			425 (190) *	12 (3) *	9 (3) *
Hospital Z			385 (190) *	8 (3) *	7 (3) *
Total			900	29	24

Note: We used “*” to mark the shared interview minutes, shared interview numbers, and shared informant numbers by these three cases.

**Table 4:** Participant observation data sources.

Cases	Observations	Observation Code	Observation Minutes	Observation N
Case 1: Hospital X	Three doctors used the voice-based AI EMR system	1OB01	60	1
	One doctor used AI-assisted medical imaging system	1OB02	20	1
	One doctor used AI-assisted diagnosis system	1OB03	20	1
	Different patients or visitors interacted with the receptionist robot	1OB04	60	1
	Two hospital service staff (such as the gateman) helped the patients on the use of receptionist robot	1OB05	30	1
	Two iFlytek employees updated the receptionist robot	1OB06	20	1
	Demonstration of AI-assisted diagnosis system that provided to visiting government officers and doctors from other Chinese hospitals, given by both doctors from Hospital X of Anhui and on-site staff from iFlytek.	1OB07	90	1
	Demonstration of all four AI systems provided by iFlytek’s vice CEO and expert guider at the exhibition hall in the iFlytek’s building.	OB01 *	80 *	1 *
Total minutes of Case 1				8
Case 2: Hospital Y	Different patients or visitors interacted with the receptionist robot	2OB01	60	1
	Demonstration of all four AI systems provided by iFlytek’s vice CEO and expert guider at the exhibition hall in the iFlytek’s building.	OB01 *	80 *	1 *
Total minutes of Case 2				2
Case 3: Hospital Z	One doctor used the voice-based AI EMR system	3OB01	30	1
	Demonstration of all four AI systems provided by iFlytek’s vice CEO and expert guider at the exhibition hall in the iFlytek’s building.	3OB02 *	80 *	1 *
Total minutes of Case 3			110	2
Total minutes of all three cases			470	10

Note: We used “*” to mark the shared observation minutes, shared observation numbers, and shared observation numbers by these three cases.

**Table 5:** Example of the interview data coding procedure.

Empirical Data	First-Order Coding	Second-Order Coding	Third-Order Coding
My leader is very active on the development of this system [AI-assisted medical imaging system]. I know they have some collaborations [between the hospital and the iFlytek]. [2HD03]	Punished by leaders	Coercive power	Non-knowledge-based
Last year [at the beginning several months], every week we have a regular meeting with doctors and hospital managers. During the meeting, head of each department from the hospital attended. And, doctors need to report the use condition of AI products during the weekly meeting. […] If they didn’t use, then they have nothing to report. […] This gives doctors pressure to use the AI product. [4iE03]	Report the usage data of AI during the department weekly meeting	Legitimate power	
At the first year, we organized marketing activities to facilitate the doctors’ and patients’ use to AI. […] We set first prize, second prize and third prize to users […] and counted by usage count and valuable feedbacks. [4iE02]	Reward doctors who are active participated on the use and development process of AI	Reward power	
In some departments [of the hospital], the using condition is much better than other departments. […] I think the reason is [in one department] there is an overall phenomenon. […] Some doctors would like to use because they saw that their colleague is using. [4iE03]	My colleague is using AI	Referent power	
I talk to them [the employee from iFlytek] very frequently. […] If I find problems, I will talk to them and ask questions in time. They can solve most of the problems. […] I would like to continue use. [1HM03]	Keep updating the system and provide expert solutions to problems faced by doctors	Expert power	Knowledge-based
Last month, we invited the AI expert Mr He to give a talk to doctors. […] During the talk, the basic knowledge about AI, for example the developing history of AI and key advantaged AI techniques and uses in different areas are introduced to doctors. [4iE02]	Encouraging knowledge sharing of AI to doctors	Informational power	
They [iFlytek] have trainings for us, especially at the beginning. […] it is very helpful. […] And anytime they have updates of the system, they will have a small-scale training for us to let us know the new functions and changes. [2HD02]	Provide training to doctors		

**Table 6:** Mapping the relationship between learning algorithm ability of AI and power structure.

Learning Algorithm Ability	Low		High	
	Receptionist Robot	Voice-Based EMR	Diagnostic AI	Medical Imaging AI
Hospital X	Informational power Expert power Referent power Coercive power	Informational power Expert power	Reward power Legitimate power	Reward power Legitimate power
Hospital Y	Informational power Referent power	Expert power		
Hospital Z		Informational power Expert power	Reward power Legitimate power	
Power structure	Knowledge-based		Non-knowledge-based	

**Table 7:** Learning algorithm ability and training data of AI.

Required Training Data Input	Receptionist Robot	Voice-Based EMR	Medical Imaging AI	Diagnostic AI
Data format/details	Text, voice, hospital background data	Text, voice, clinical examination-related data	Text, image, clinic case-related data	Text, image, voice, clinic case-related data
Data volume	Small	Small	Large	Large
Data velocity	Fast	Fast	Slow	Slow
Data quality	High	High	Low	Low
Data diversity	Low	Low	High	High
Level of learning algorithm ability	Low	Low	High	High
